# Possible strategies to cross the blood–brain barrier

**DOI:** 10.1186/s13052-018-0563-0

**Published:** 2018-11-16

**Authors:** Cinzia M. Bellettato, Maurizio Scarpa

**Affiliations:** 1 Brains For Brain Foundation, Padova, Italy; 2 European Reference Network for Hereditary Metabolic Diseases, MetabERN, Wiesbaden, Germany; 3 Department of Child and Adolescent Medicine, Center for Rare Diseases, Helios Dr. Horst Schmidt Kliniken, Ludwig-Erhard-Straße 100, 65199 Wiesbaden, Germany; 4 Department for the Woman and Child Health, University of Padova, Padova, Italy

**Keywords:** Mucopolysaccharidoses, Blood–brain barrier, Central nervous system, Drug delivery

## Abstract

The mucopolysaccharidoses (MPS) are a heterogeneous group of in-born metabolic conditions caused by genetic defects that result in the absence or severe deficiency of one of the lysosomal hydrolases responsible for the degradation of glycosaminoglycans (GAGs). Such enzyme deficiency causes accumulation of GAGs that begins in infancy and progressively worsens, often affecting several organs including the central nervous system (CNS) inducing mental retardation, progressive neurodegeneration, and premature death. Over the last years, enormous progress has been made in the treatment of many MPS types, and available treatments are efficacious for many of them. Nevertheless, treatment of MPS with CNS involvement is limited mostly because of delivery impediments related to the presence of the blood–brain barrier (BBB). This chapter presents an overview of the BBB and of the different strategies that have been developed to overcome the problem of drug transport at the BBB, assuring efficient delivery of therapeutic agents to the brain.

## Background

The mucopolysaccharidoses (MPS) are a heterogeneous group of hereditary metabolic disorders caused by genetic defects that result in the absence or severe deficiency of one of the specific lysosomal enzymes involved in the degradation of mucopolysaccharides, also known as glycosaminoglycans (GAGs). Such deficiency leads to an abnormal accumulation of GAGs in various organs and tissues including the arteries, skeleton, eyes, joints, ears, skin, liver, spleen, and/or teeth. Storage may also be found in the respiratory system, blood, and bone marrow. MPS mostly affect the paediatric population and unfortunately about 70% of all affected patients present a neurological involvement with storage also affecting the central nervous system (CNS), where GAG accumulation can seriously affect neurons leading to death through apoptosis or necrosis during the advanced stages of the disease [1]. Although sharing many clinical features, MPS manifest a wide heterogeneity in their severity within the same phenotype, with a wide spectrum of clinical forms ranging from attenuated (slowly progressing) to very severe (rapidly progressing) forms of the disease. CNS pathology typically causes mental retardation, progressive neurodegeneration, and premature death. In particular, and depending on the MPS subtype, affected individuals may have normal intellect or may be profoundly impaired, they may experience developmental delay, or they may have severe behavioural problems. Severe brain involvement typically characterizes all forms of MPS III, while in MPS I, II, and VII a progressive mental delay accompanied by behavioural problems affects the severe forms of these diseases. MPS IV and VI do not commonly show any significant brain involvement.

Although there is no curative treatment for MPS, today several therapeutic options for MPS exist and are commercially available including enzyme replacement therapy (ERT). ERT consists of replacing the defective enzyme thorough regular intravenous infusions of a working copy of the enzyme which is then scavenged by affected cells through the mannose-6-phosphate (M6P) pathway, then endocytosed and translocated into the lysosomes where they replace the defective enzyme and restore functional activity. However, improvement in the brain is limited by the presence of the blood–brain barrier (BBB), a selectively permeable barrier between the capillaries and the brain that prevents the efficient transfer of high molecular weight therapeutic drugs from the blood to the brain parenchyma and thus hinders effective treatment of MPS with CNS involvement. The greatest challenge therefore consists of developing therapies capable of achieving efficient delivery of the recombinant enzyme in the CNS across the BBB. It must be highlighted that not only patients with MPS but, in general, more than 70% of all patients affected by lysosomal storage disorders (LSDs) suffer from different grades of CNS involvement. Research efforts are therefore today particularly focused on the development of new strategic approaches for enhancing enzyme delivery across the BBB.

## Blood–brain barrier and drug delivery

**Fig. 1:**
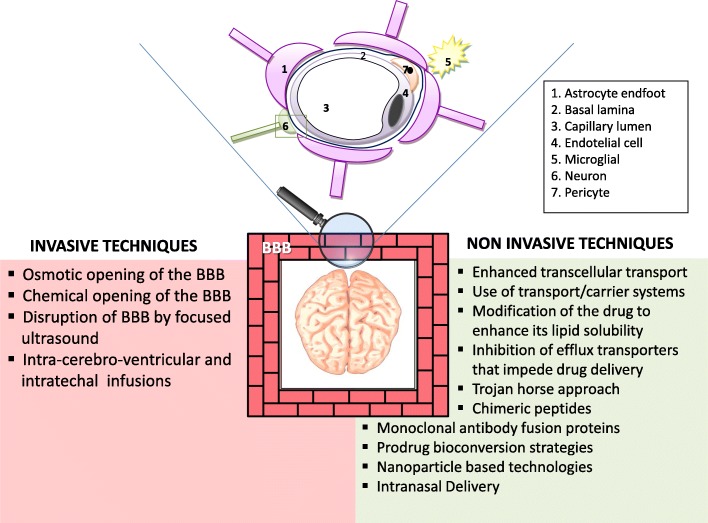
Angioarchitecture of the blood–brain barrier and techniques for brain drug delivery

The BBB is a semi-permeable membranous barrier, localized at the interface between the blood and the cerebral tissue, composed of a complex system of endothelial cells, astroglia, pericytes, and perivascular mast cells (Fig. 1). It is mainly responsible for rigorously controlling the exchanges between the two compartments allowing only certain molecules or ions to pass through by diffusion or occasionally by more specialized processes of facilitated diffusion, passive transport, or active transport. It is thus responsible for creating and maintaining homeostasis for neuronal functions, defending the system against toxic insults, regulating the communication between the periphery and the CNS, and providing the brain with nutrients.

This is achieved through: 1) prevention of the paracellular diffusion of hydrophilic compounds; 2) mediation of the active transport of nutrients to the brain; 3) activation of efflux transport of hydrophobic molecules and drugs from the brain to the blood; and 4) regulation of the transendothelial migration of circulating blood cells and pathogens.

This unique dynamic cellular complex is characterized in the BBB by the fact that the endothelial cells fit tightly together giving origin to a continuous endothelium which is not fenestrated, and which exhibits a relatively low endocytic activity; this differs from the other parts of the body. The paracellular aqueous diffusional pathways between the cells is also further prevented by the presence of tight junctions (TJs) among adjacent endothelial cells. Thanks to their adhesive function, TJs effectively seal microvessels and preclude the passive diffusion of polar solutes and proteins in and out of the CNS. Thanks to such restrictive angioarchitecture, the BBB finely regulates and controls the internal brain compartment by constraining any molecular traffic to be primarily across the cell (transcellular), thus deeply restricting the passage of drugs and other undesired solutes [2].

Being the tightest endothelium in the body, the BBB also represents the main impediment to drug delivery to the brain. For this reason, CNS drug development is devoted to both CNS drug discovery and CNS drug delivery. In particular, the understanding of molecular and physiological mechanisms involved in the transport of compounds through the BBB represents an important key for brain delivery.

## Transport at the BBB

Generally, only lipid soluble (lipophilic) molecules with a low molecular weight (under 400–600 Da) and of positive charge can cross the BBB. Other molecules require certain cell endogenous transport systems, such as carrier-mediated transport, receptor-mediated transport, or absorptive-mediated transport. Commonly, there are five basic mechanisms by which solute molecules move across membranes.

Firstly, simple (or passive) diffusion is a spontaneous process depending on the magnitude of the concentration gradient. Several lipid-soluble molecules can enter the cell membrane and diffuse passively across the endothelium into the brain. There is a correlation between increased lipid solubility and the rate and extent of penetration into the brain. Blood gases and several drugs, for example anaesthetics and heroin, enter the brain in this fashion [3].

Secondly, the solute carriers (SLC) constitute a superfamily of membrane transport proteins that facilitate the bi-directional movement of solutes across the cell membrane. Polar molecules may be transported across the endothelial cell membrane. Differing from their ATP-binding cassette (ABC) transporter counterparts, SLC transport does not require ATP since it is driven either by electrochemical gradients (i.e. Na^+^ or H^+^ gradient) or by concentration gradients established by the solutes that are being transported. SLC are therefore classified as either facilitated transporters or secondary active transporters [4].

Typically, the entry into the brain of major nutrients such as glucose, amino acids, nucleosides, monocarboxylates, and organic anions and cations, and the efflux of several metabolites are mediated by SLC. Some drugs (e.g. l-DOPA) are also transported into the brain by these transporters [5, 6].

Thirdly, carrier-mediated efflux (efflux transporters) represents another significant transport mechanism at the BBB. ABC transporters have a wide affinity for a wide category of solutes, especially large, lipid-soluble molecules with a number of nitrogen and oxygen atoms in their structure. These ABC transporters use ATP hydrolysis to pump molecules across the membrane and therefore they can force the efflux of solutes against a concentration gradient. P-glycoprotein (Pgp:ABCB1) and breast cancer-related protein (BCRP:ABCG2) are the principal ABC efflux transporters in the BBB. A number of cytotoxic drugs are substrates, which confounds the treatment of brain tumours and brain metastases [7].

Fourthly, receptor-mediated transcytosis is a class of transport system that utilizes the vesicular transport system of the endothelial cells to transport substrates on the brain side of the barrier. Receptor-mediated transcytosis (RMT) is commonly induced by the binding of large molecules such as peptides and proteins to receptors (insulin receptor, transferrin receptor, low-density lipoprotein (LDL) receptor its related protein, etc.) that are highly expressed on the endothelial cell membrane. Typically, nutrients such as iron, insulin, and leptin are transported into the brain by such an endocytic event also known as transcytosis [8, 9].

Finally, there is diapedesis of mononuclear leukocytes. Leukocytes may penetrate the BBB by transendothelial diapedesis, migrating directly through the cytoplasm of the endothelial cells without tight junction disruption. Once in the brain they become microglia, the immune competent cells of the brain. It has been speculated that, during the brain inflammatory process, leucocytes can also move additionally through the tight junctions and infiltrate at a faster rate [10].

The absence of mannose or mannose-6-phosphate receptor expression at the luminal cell membrane of the BBB, the absence of a BBB transport system for acid hydrolases, and the high molecular weight of the compounds render any paracellular or transcellular diffusion of systematic infused therapeutic enzymes across the BBB almost non-existent. This results in the incapacity of enzymes infused by ERT to cross the barrier and enter the CNS in any significant amount.

It has been estimated that more than 90% of all small-molecule drugs and nearly 100% of all larger therapeutics are not able to overcome the BBB [11]. Huge research efforts are therefore directed to develop new strategies capable of effectively crossing the BBB and delivering therapeutic products in the CNS. Several routes of administration are under investigation, and many research attempts are being made to modify the drug physicochemical properties to promote their related permeability across the BBB, thus allowing CNS brain targeting.

These strategic therapeutic approaches for bypassing the BBB can be broadly classified into one or more of the following categories: invasive techniques and non-invasive or miscellaneous techniques (Fig. 1).

### Invasive techniques

#### Blood–brain barrier transient disruption

This technique consists of the use of noxious agents, hyperosmotic solutions, or ultrasounds (mannitol, dimethyl sulphoxide, ethanol, metals, glycerol and polysorbate-80, X-irradiation, etc.) to shrink the brain’s endothelial cells by breaking down tight junctions, allowing various molecules to pass into the cerebral tissue. Unfortunately, this technique has several limitations; it is in fact non-patient friendly, and can compromise the integrity and the physiological functions of the BBB leading to potential accumulation of unwanted blood components, neurotoxic, xenobiotics, and exogenous agents, thus causing injury to the CNS.

#### Intracerebroventricular and intrathecal infusion

These strategies consist of the injection or intraventricular infusion of therapeutic proteins directly into the cerebrospinal fluid (CSF).

The advantages of these methods over systemic ERT are that they allow delivery to the brain of a higher amount of enzymes and, consequently, it is not necessary to use massive concentrations of therapeutic drugs. Furthermore, these strategies overcome the problems related to the short half-life of drugs in the blood, avoiding the ones related to systemic exposure and toxicity [8]. Intrathecal drug administration can be accomplished by lumbar puncture or by an implanted intrathecal drug delivery device (IDDD).

Data from animal models of MPS I, II, and IIIA, and also of other LSDs such as infantile neuronal ceroid lipofuscinosis and Niemann-Pick A, indicate that ERT through intrathecal injection is able to distribute the recombinant enzyme throughout the CNS where it can penetrate the brain tissue promoting the clearance of accumulated material within the lysosomes [12, 13]. In the past few years, thanks to the availability of mouse and dog models capable of recapitulating the MPS IIIA neuropathological features, research efforts have been particularly focused on the development and testing of new therapies for brain involvement in MPS IIIA. Following the encouraging results obtained from animal studies showing that repeated direct infusion of a missing enzyme via cerebrospinal fluid injection is an effective treatment for pathological changes in the brain of mice and dogs [14], clinical trials have been initiated in humans to test the safety and tolerability of recombinant human heparan-N-sulfatase (rhHNS) administered via IDDD in patients with MPS IIIA (NCT01155778 and NCT01299727) [15, 16]. Similarly, the safety of idursulphase formulated for intrathecal administration (idursulphase-IT) via IDDD has been tested on MPS II patients [17]. Although outcomes from these studies encourage further investigational studies, the clinical application of these approaches is considered challenging due to the short half-life of the enzymes. To improve efficacy and increase the chance for clinical success, repeated administrations are necessary with an increased risk of toxic effects [16].

### Non-invasive techniques

Non-invasive techniques mainly consist of pharmacological strategies capable of modifying drugs to facilitate transport across the BBB. The main non-invasive techniques are discussed below.

#### Modification of the drug to enhance its lipid solubility

Since lipid solubility is a strategic factor in passive diffusion into the BBB, this technique consists of chemically transforming water-soluble molecules into lipid-soluble molecules capable of crossing the BBB. This is performed by adding lipid groups or functional groups to the polar ends of drug molecules [18].

#### Use of transport/carrier systems

This technique consists of chemically modifying a small-molecule therapeutic drug to allow it to use the endogenous transport/carrier systems, mimicking the structure of the related specific endogenous molecules (monosaccharides, monocarboxylic acid, large neutral amino acids, basic amino acid, acidic amino acids, amines, purine bases, nucleosides, vitamins, and hormones). The glucose transporter type 1 (GLUT1), the large neutral amino-acid transporter type 1 (LAT1), the cationic amino-acid transporter type 1 (CAT1), the monocarboxylic acid transporter type 1 (MCT1), and the equilibrative nucleoside transporter 1 (ENT1) are some of the most commonly endogenous carrier-mediated BBB transporters used as carrier systems employed for drug delivery [19].

#### Inhibition of efflux transporters that impede drug delivery

This technique consists of the pharmacological inhibition of selected efflux transporters that, being expressed by the cerebrovascular endothelium, prevent blood-to-brain drug uptake. Examples are P-glycoprotein, breast cancer resistance protein (BCRP) in humans, Bcrp in rodents, and multidrug resistance proteins (MRPs in humans; Mrps in rodents), etc. [4].

#### Trojan horse approach

This method consists of using molecules such as endogenous ligands or monoclonal antibodies that, acting as molecular ‘Trojan horses’, bind exofacial epitopes on BBB receptor-mediated transport systems, triggering internalization of the receptor and of the attached drug. After internalization, the two components separate and take distinct paths, with the receptor travelling back to the membrane and the therapeutic drug freely diffusing into the brain parenchyma. This technique is being used to ferry drugs, proteins, and non-viral gene medicines across the BBB [4].

#### Chimeric peptides

This technique is being used for the transport across the BBB of those therapeutic compounds that are transportable only at a very low rate. This method consists of making a chimeric peptide by covalently coupling an otherwise non-transportable drug to a BBB-transportable peptide vector (e.g. cationized albumin, insulin, transferrin, etc.) by a disulphide bond. Such a chimeric peptide is then endocytosed by the capillary endothelial cells through receptor-mediated transcytosis and transported to the brain where, thanks to the presence of brain disulphide reductases, the pharmacologically active therapeutic peptide can be cleaved from the peptide vector. BBB peptide receptor systems include those for insulin, insulin-like growth factor, transferrin, and leptin. The brain contains the necessary disulphide reductases for rapid cleavage of the chimeric peptide [20].

#### Monoclonal antibody fusion proteins

This technique consists of re-engineering the biologic drug as an IgG fusion protein, thus making brain penetration possible. The IgG domain, being a monoclonal antibody (mAb), binds an endogenous BBB receptor such as the transferrin receptor (TfR). The complex, referred to as TfRmAb, acts as a molecular Trojan horse and delivers the attached drug compound into the brain via receptor-mediated transport on the endogenous BBB TfR [21]. The mAbs are also used as a transport vector for the brain delivery of genes [22]. One mAb against the insulin receptor has proven to be one of the most potent transport vectors. The BBB molecular Trojan horse technology allows the re-engineering of a widespread range of recombinant protein therapeutics for targeted drug delivery to the brain [23]. Studies aimed at assessing the efficacy of this technology are ongoing in mouse models of neurological diseases, including Parkinson’s disease, stroke, Alzheimer’s disease, and lysosomal storage disorders [21].

#### Pro-drug bioconversion strategies

These methods consist of developing pro-drug compounds (also called pro-agents) that are therapeutically inactive agents capable of crossing the BBB and entering into the brain parenchyma. Once reaching the target site they undergo enzymatic and/or chemical transformations and modify their structure achieving a biologically active form able to exert the desired pharmacological effect [24].

#### Nanoparticle-based technologies

This approach is mainly based on the use of nanosized technology for drug release in the brain. This delivery system uses a wide variety of nanoscale drug delivery platforms mainly including lipid- and polymer-based nanoparticles (NPs) that assure a controlled and improved release of their cargo by protecting loaded drugs from being metabolized [25]. The main efforts today are mainly focused on increasing the ability of NPs to effectively target the therapeutic site, thus minimizing the doses of drugs released at undesired sites [26]. Considering that NPs have shown to be effective drug carriers, the feasibility of using them for enabling a more effective delivery to organs has been investigated using laronidase surface-functionalized lipid-core nanocapsules (L-MLNC) for the treatment of MPS I. Outcomes show that L-MLNC are able to modify drug biodistribution with important repercussions, including the possibility of reducing the dosage and thereby enhancing ERT efficiency and/or reducing the cost [27]. Another recent study has addressed the feasibility of loading arylsulfatase B onto poly(butyl cyanoacrylate) (PBCA) nanoparticles to affect neurological manifestations such as spinal cord compression in MPS VI by delivery of therapeutic enzyme across the BBB [28]. Recently, in-vivo and in-vitro studies using polylactide-co-glycolide (PLGA) NPs conjugated with a simil-opioid glycopeptide (g7) have proved that these NPs are able to overcome the BBB at a level of up to 10% of the injected dose [29]. Moreover, it has been demonstrated that g7-NPs, thanks to the intra- and intercellular vesicular transport, can target specific cells in the brain and are also able to reach the CNS by intraperitoneal, intranasal, and oral administrations [30]. These results have encouraged the application of g7-NPs for delivering therapeutic enzymes across the BBB, as performed in a recently published study in two murine models for MPS I and MPS II aimed at assessing the g7-NP brain delivery capacity using a model drug (FITC-albumin) with a high molecular weight, similar to the therapeutic enzymes commonly used in ERT protocols [31]. Results showed the g7-NPs ability to cross the BBB and to widely localize in all brain parenchyma, demonstrating the applicability of NPs for therapeutic brain delivery of high molecular weight molecules and thus encouraging their potential application to enzyme delivery to the brain [31].

#### Gene therapy

This technique consists of transferring recombinant DNA with therapeutic function directly into the cells of specific organs. It represents a promising solution for those neurodegenerative conditions where the neuropathology is spread throughout the entire brain and therefore a global CNS gene delivery is required for an effective treatment [32]. Two types of different applications are possible: “ex vivo”, in which the target cells are collected, treated by genetic engineering techniques, and re-infused into the patient; and “in vivo”, in which the gene is directly transferred into the body via a suitable vector such as plasmids or non-pathogenic viral vectors (retrovirus, adenovirus, adeno-associated virus (AAV)) [8]. Extensive studies of intravenous vector administration have been conducted in new-born mice models of MPS I and MPS VII, showing encouraging results [33]. However, when translated to adult animal models, problems with immune response were encountered; although the use of systemically delivered AAV vectors in murine models of MPS I, IIIA, IIIB, and VI has demonstrated a reduction in the corresponding substrates in the CNS [34], it remains difficult to translate these achievements to larger animal models because of differences in biology, anatomy, and size. These differences make it necessary to accurate scale the dosage, and efficacy and toxicity tests due to their longer life-span. For all these reasons, intracerebral gene therapy can open up a new horizon in this field.

#### Intracerebral gene therapy

The technique consists of directly injecting viral gene transfer into the brain parenchyma or ventricular system. Studies in certain small (rodent) and large (canine and feline) animal models of LSDs have shown functional improvement and reduction in lysosomal storage [35]. Various non-neurotropic viral vectors have been studied for in-vivo CNS gene transfer in the brain of MPS I, MPS IIIA, MPS IIIB, and MPS VII mouse models via AAV, adenovirus, and lentivirus [36]. Interesting outcomes from phase I/II clinical trial aimed at evaluating the feasibility and safety of brain injection of AAV vector for MPS IIIA and IIIB suggest that intraparenchymal delivery may constitute a realistic option for neuropathic MPS [37]. Direct delivery into the CNS through CSF injections of various AAV serotypes has also been tested in animal models of MPS I, IIIA, IIIB, and VII, but further studies are required to confirm the benefits of this approach [37].

#### Intranasal drug delivery

This approach constitutes a potential alternative method for therapeutic brain targeting and is based on the direct transport into the CSF enabled by the presence of the neural pathways connecting the nasal mucosa and the brain [38]. Such a nose-to-brain pathway allows rapid delivery of the therapeutic molecules to the CNS within minutes, bypassing the BBB. Absorption occurs by transcellular and paracellular passive absorption, carrier-mediated transport, and absorption through transcytosis.

Lipid-based NPs have been studied for intranasal drug delivery and this non-invasive strategy approach has been used to assess the efficacy of treatment of neurological manifestation in MPS I disease. Laboratory experiments in mice showed that intranasal administration of an α-l-iduronidase (IDUA) encoding adeno-associated virus serotype 9 (AAV9) vector results in enzyme diffusion into deeper areas of the brain and related reduction of tissue GAG storage materials in the brain [39]. Intranasal delivery could then potentially be used to treat CNS manifestations of MPS I. Nevertheless, several restrictions for its use exist, such as the existence of upper limits of the concentrations that can be achieved in different regions of the brain and spinal cord, the reduction of the drug delivery efficiency with increasing molecular weight of the drug, and large variability in nasal absorption caused by irritation of the nasal mucosal or common nasal pathology, such as with the common cold, etc. [38].

## Conclusion

Treatment of MPS is mostly challenging because delivery of therapeutic drug molecules to the brain is frequently obstructed by the presence of the BBB which constitutes the main obstacle for the treatment of those forms of MPS characterized by neurological involvement. It is clear that nowadays there is an urgent need to direct research efforts to overcome the BBB and to develop new therapeutic strategies capable of successfully targeting the drug to the brain compartment. Although there are some interesting outcomes at present for brain-targeted drug delivery by mean of clinical (invasive) or technological (non-invasive) approaches using innovative drug delivery systems for crossing the BBB, these are still not well defined. Further studies are needed to better understand the BBB transport systems, to assess brain drug pharmacokinetics, and to improve the delivery and distribution of the drugs into specific areas of the brain. Nevertheless, it seems quite reasonable to think that the BBB is no longer an impenetrable barrier. This raises significant hopes, not just for patients affected by MPS but for all those suffering from LSDs and other conditions characterized by brain pathology.

## Abbreviations

AAV: Adeno-associated virus
ABC: ATP-binding cassette
BBB: Blood–brain barrier
CNS: Central nervous system
CSF: Cerebrospinal fluid
ERT: Enzyme replacement therapy
g7: Simil-opioid glycopeptide
GAG: Glycosaminoglycan
IDDD: Intrathecal drug delivery device
LSD: Lysosomal storage disorder
mAb: Monoclonal antibody
MPS: Mucopolysaccharidosi(e)s
NP: Nanoparticle
SLC: Solute carriers
TfR: Transferrin receptor
TJ: Tight junction
